# Recent Advances in *Panax ginseng* C.A. Meyer as a Herb for Anti-Fatigue: An Effects and Mechanisms Review

**DOI:** 10.3390/foods10051030

**Published:** 2021-05-10

**Authors:** Guanyu Lu, Zhuoting Liu, Xu Wang, Chunling Wang

**Affiliations:** State Key Laboratory of Food Nutrition and Safety, College of Food Science and Engineering, Tianjin University of Science and Technology, Tianjin 300457, China; lgy15253119716@163.com (G.L.); lzt1215524176@163.com (Z.L.); wangchunling@tust.edu.cn (C.W.)

**Keywords:** *Panax ginseng*, anti-fatigue, animal models, clinical

## Abstract

As an ancient Chinese herbal medicine, *Panax ginseng* C.A. Meyer (*P. ginseng*) has been used both as food and medicine for nutrient supplements and treatment of human diseases in China for years. Fatigue, as a complex and multi-cause symptom, harms life from all sides. Millions worldwide suffer from fatigue, mainly caused by physical labor, mental stress, and chronic diseases. Multiple medicines, especially *P. ginseng*, were used for many patients or sub-healthy people who suffer from fatigue as a treatment or healthcare product. This review covers the extract and major components of *P. ginseng* with the function of anti-fatigue and summarizes the anti-fatigue effect of *P. ginseng* for different types of fatigue in animal models and clinical studies. In addition, the anti-fatigue mechanism of *P. ginseng* associated with enhancing energy metabolism, antioxidant and anti-inflammatory activity is discussed.

## 1. Introduction

With the increasing pace of modern life, many unhealthy lifestyles, such as unbalanced diet, irregular work and rest, depressed mood, are becoming more widespread. All of these can lead to a sub-healthy state with unexplained fatigue. Fatigue often leads to anxiety and depression. It is related to cognitive impairment, sleep quality, physical dysfunction, and energy balance [1]. Long-term or severe fatigue may also increase the incidence of diseases related to the immune system [2], such as aging, multiple sclerosis, and Parkinson’s disease, which is seriously harmful to the work and life of patients [3]. In addition, diabetes, liver diseases, cancer, and some other diseases may also cause fatigue symptoms, also called disease-related fatigue [4]. Normal fatigue occurring after strong physical effort can be alleviated by rest or lifestyle change [5]. However, pathological fatigue cannot improve with rest [6]. Fatigue, as a complex and pathologically unknown physiological phenomenon, has seriously threatened human health. A single-component chemical may lead to some side effects in the long-term medication process [1]. Medicine food homology (MFH) materials not only hold nutritional value but also function in the prevention and treatment of diseases [7]. *Panax ginseng* C.A. Meyer (*P. ginseng*) is a kind of Acanthopanax herb and is considered an MFH material [8]. As an ancient Chinese herbal medicine, *P. ginseng* has been used for over 4000 years to remain healthy or treat diseases in China.

In many countries, especially east Asian countries such as China, Korea, and Japan, people believe that *P. ginseng* is the king of herbs because of its long history and various pharmacological activities. The diverse pharmacological activities of ginseng are determined by its complex and large amount of ingredients [9,10,11]. Nowadays, *P. ginseng* is received increasing attention as a kind of anti-fatigue product with obvious efficacy and fewer side effects. To date, the study of the anti-fatigue effect of *P. ginseng* involves different components of *P. ginseng*, such as some types of ginsenosides and ginseng polysaccharides, which pointed at different mechanisms as well as different types of fatigue. We searched “Panax ginseng” and “anti-fatigue” as keywords in PubMed and focused on the anti-fatigue studies of *Panax ginseng* C.A. Meyer. This review covers animal and human studies after 2010 with diverse materials of *P. ginseng*, such as mixture extract or some monomer compounds. The discovered anti-fatigue mechanisms of ginseng include regulation of energy metabolism, reduced metabolite accumulation and antioxidant and anti-inflammatory activity.

## 2. Biomarkers and Main Mechanisms Involved in Anti-Fatigue Activity of *P. ginseng*

**Figure 1:**
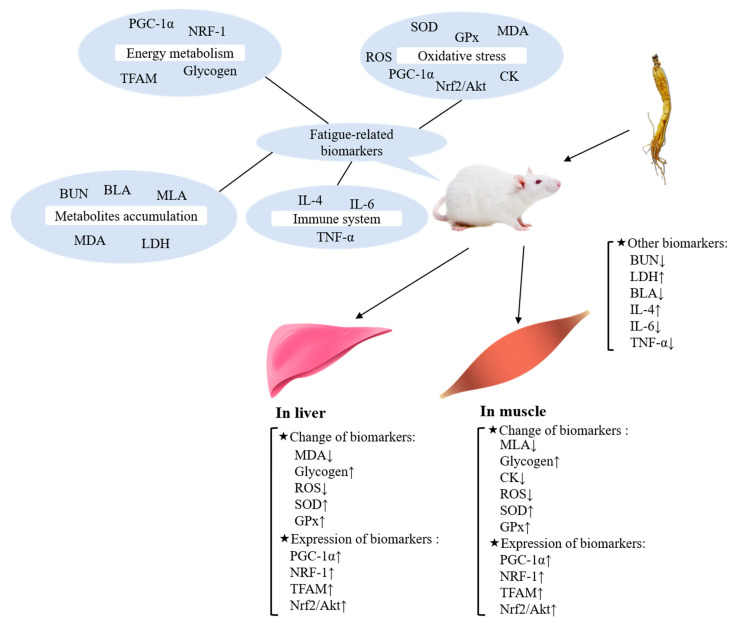
Major fatigue-related biomarkers and their mechanisms.

As a symptom with complex mechanisms, the occurrence of fatigue causes various changes of several biomarkers in organisms (see Figure 1).

When it comes to fatigue, the immediate reaction is energy deficiency. When energy has been depleted, sensations of fatigue and exhaustion occur in an organism. Peroxisome proliferator-activated receptor-gamma coactivator 1alpha (PGC-1α) is a factor, which can affect the energy metabolism with multiple pathways [1]. For example, PGC-1α has been found as a molecular trigger that stimulates energetic food intake, fatty acid metabolism and activates the expression of other genes related to lipid metabolism [12]. In addition, PGC-1α also plays a critical role in organism glucose and energy homeostasis [13]. The promoted energy metabolism provides ample energy for improving exercise performance and alleviates or delays fatigue. As the main energy source for organisms, glycogen content in the liver and muscle directly impacts exercise and fatigue symptoms [14]. Moreover, NRF-1 (a target of PGC-1α [15]) and TFAM (a crucial protein of mitochondrial DNA replication and transcription [16]) are two positive key regulators of mitochondrial biogenesis and function that can affect the energy supply of mitochondria [17,18].

The occurrence of fatigue affects the organism’s metabolism, which causes the accumulation of metabolites. Because of the increased energy requirement, protein and amino acids in the organism decompose and release metabolites. Blood urea nitrogen (BUN) is one of the products of protein and amino acid degradation, which can reduce endurance and cause fatigue [19]. Blood and muscle lactic acid (BLA, MLA) produced during exercise can cause fatigue by reducing the pH in blood and muscle tissue, which is considered a major trigger of fatigue symptoms [20,21]. In addition, increased LDH activity can accelerate the clearance of lactic acid to relieve fatigue.

Oxidative damage in energy-supplying organs causes tissue damage and impedes energy supplementation, finally leading to fatigue. Reactive oxygen species (ROS) is considered one of the factors causing oxidative stress response and subsequent fatigue symptoms because ROS can trigger the lipid peroxidation of mitochondrial membrane and damage mitochondrion [22]. Concerning creatine phosphokinase (CK), it is reported that CK is one of the indirect indexes of membrane structure damage [23]. Malondialdehyde (MDA), the metabolite of lipid peroxidation, is a symbolic indicator of oxidative stress in the human body. Furthermore, previous studies have proved that inhibition of oxidative stress can relieve fatigue [24,25]. As the enzymes involved in oxidative stress response, glutathione peroxidase (GPx), superoxide dismutase (SOD), and catalase can participate in the decomposition of ROS into H_2_O and O_2_ [12], which can reduce bodily injury caused by oxidative stress responses. Another major mechanism for cells defending against oxidative stress is the activation of the Nrf2-ARE signaling pathway, which controls the expression of genes involved in removing ROS [26,27]. It also has been found that PGC-1α loss leads to exercise endurance decline and multiple tissue dysfunction in oxidative metabolisms, such as liver and muscle [28].

In addition to the above impactors of fatigue, pathological fatigue leads to the excessive release of proinflammatory cytokines such as IL-6, TNF-α, and IL-4 [29]. It has been reported that the secretion of cytokines is closely related to the mice immune regulation, which can affect the fatigue state of the body [30]. Some diseases like cancer and its treatment process can exacerbate the release of peripheral proinflammatory cytokines and physical deterioration, leading to the occurrence of acute fatigue symptoms.

## 3. Anti-Fatigue Effect of *P. ginseng* on Animal Models

Pharmacological studies of natural products must be based on animal experiments [31]. To date, the extent of fatigue in animal models is generally estimated by behavioral tests such as the forced swimming test, the forced running test, the rotarod test, and the forelimb grip test because of their visibility and ease of operation. Besides the behavioral test, several biomarkers mentioned in Section 2 are also used for evaluating fatigue extent. The anti-fatigue effects of mixture extract and monomer compounds in *P. ginseng* are summarized in Table 1 and Table 2.

### 3.1. The Anti-Fatigue Effects of P. ginseng Extract

#### 3.1.1. In Normal Fatigue

Kai Xin San (KXS), recorded in “*Bei Ji Qian Jin Yao Fang*” (An ancient book of Traditional Chinese Medicine) in the Tang Dynasty in China, was composed of *P. ginseng* and some other kinds of traditional Chinese medicines, including *Acorus tatarinowii*, *Poria cocos* and *Polygala tenuifolia*. KXS can cure symptoms like desolation, moodiness, forgetfulness, etc., which are similar to the neuroses, such as depression, anxiety, and learning and memory impairments [49]. Hu et al. indicated that KXS exhibits anti-fatigue activity reflected in the effects on several biomarkers for fatigue [41], including the levels of blood urea nitrogen (BUN), testosterone (T), and lactate dehydrogenase (LDH) activity in serum, glycogen level in liver and muscle, the content of blood lactic acid (BLA) and malondialdehyde (MDA), β-endorphin level in the brain and the activity of superoxide dismutase (SOD). Mild androgen deficiency may account for increased fatigue, and the improvement of testosterone can reverse the increased fatigue [50,51]. In addition, a lower level of β-endorphin has also been demonstrated to be beneficial to endurance exercise [52]. The results showed that after KXS treatment, the increased β-endorphin and MDA levels were inhibited, and the decreased SOD activity was reversed. It has been suggested that KXS might improve the metabolic control of exercise and the activation of energy metabolism to show the anti-fatigue effect. Increased SOD activity and reduced lipid oxidation indicate that KXS has antioxidant activity, and it can protect the corpuscular membrane most likely by preventing lipid oxidation.

Previously, Wang et al. confirmed the effect of ginseng polysaccharides on normal fatigue [32]. In this study, experiments were carried out in all types of water-soluble ginseng polysaccharides, including neutral water-soluble polysaccharides (WGPN), acidic water-soluble polysaccharides (WGPA), and mixed water-soluble polysaccharides (WGP). In forced swim tests, polysaccharides can reduce immobility times. However, only WGPA can achieve the same effect at a lower dose. The forced swim test increased MDA levels and decreased glucose levels with increased LDH and CK activity and decreased GPx activity in mice serum. WGP can block all the changes, but WGPN can only reduce the change of LDH, MDA, and GPx. Importantly, WGPA can also block these effects in lower doses compared with the other polysaccharides. Additionally, triglyceride (TG) level decreased, and the activity of SOD increased after WGP and WGPA treatment. The change of LDH, MDA, and GPx suggested that ginseng polysaccharides can relieve fatigue probably by preventing lipid oxidation via modifying several enzyme activities [53]. Moreover, there is another theory that suggests that ginseng polysaccharides can regulate TG metabolism during exercise, as shown by decreasing TG levels and coinstantaneous increasing GLU levels. However, the study acknowledged that further experiments were needed to identify the mechanism of ginseng polysaccharides on regulating lipid metabolism. Moreover, the results proved that the acidic polysaccharide has higher potency to induce an anti-fatigue activity than the neutral polysaccharide.

Changbai mountain ginseng (CMG), as a kind of *P. ginseng* growing at the elevations of 2000 m or higher with major compound ginsenoside Ro, had been proven to have good anti-fatigue activity [33]. After four weeks of CMG supplementation, the mice had a stronger forelimb grip and longer endurance swimming times, which indicated that CMG might improve the exercise endurance of mice. After exercise, CMG supplementation groups had lower BLA, serum ammonia, BUN levels, and CK activity, as well as higher GLU levels. In addition, CMG supplementation groups had more glycogen storage in the liver and muscle. The increased abundance and translocation of glucose transporter type 4 (GLU4) can lead to a higher glucose level in CMG supplementation groups by a pathway that is not dependent on insulin. Accumulation of ammonia in the blood and brain during exercise can negatively affect the central nervous system and cause fatigue. Fortunately, CMG may reduce ammonia accumulation in the blood. Simultaneously, analysis of some other health-related biomarkers, such as aspartate aminotransferase (AST), alanine aminotransferase (ALT), uric acid (UA), and total cholesterol (TC), indicated that CMG had no adverse effects on major organs, such as the liver, skeletal muscle, heart, kidney, and lung. All the results suggested that the CMG extracts may improve exercise endurance and reduce body fatigue with highly edible security.

Sometimes, a combination of natural products is more effective for fatigue resistance [54]. An et al. observed the synergistic anti-fatigue effect of *P. ginseng* and acanthopanax extracts in mice [40]. After 30 days of administration, the mice in the middle-dose (about 0.3 g/(kg × d) ginseng extract and 0.25 g/(kg × d) acanthopanax extract) and high-dose (about 0.6 g/(kg × d) ginseng extract and 0.5 g/(kg × d) acanthopanax extract) groups showed better performance in the forced swim test. Mice in the middle-dose group could swim longer than those in the high-dose group. BUN content was significantly decreased, while GPx, SOD activities, and liver glycogen content were increased in the middle-dose group compared with the non-administration group. In addition, LDH, GPx and SOD activities were significantly increased in the high-dose group compared with the non-administration group. In addition to these changes, the BLA content in the high-dose group only showed a decreasing trend. The results demonstrated that combined extracts of *P. ginseng* and acanthopanax might relieve fatigue by reducing the accumulation of exercise metabolites and free radicals. It was also suggested in the study that CK as an anti-fatigue index was not so sensitive because the CK activity showed no significant difference in any group with extract treatment. However, all these viewpoints needed to be further explored. Besides the acanthopanax, some other studies showed that *P. ginseng* combined with notoginseng or *Antrodia camphorata* can also produce significant anti-fatigue effects in mice [3,39].

Ginseng oligopeptides (GOP) consisting of several amino acids such as aspartic acid, glutamate, glycine, tyrosine, histidine and serine had positive effects against normal fatigue [34]. The best performance in the forced swimming test was found in the high-dose group (500 mg/kg). Compared to low-dose and middle-dose groups, this group had increased LDH, SOD, catalase activity and liver glycogen levels and decreased BUN and MDA levels. GOP not only can inhibit the production of BLA after forced swimming tests but also can decrease the content of BLA more quickly. In addition to the change of these biomarkers, the mRNA expression of NRF-1 and TFAM was markedly increased after GOP treatment, which indicated that GOP might suppress oxidative stress by improving mitochondrial function in skeletal muscles to generate more ATP energy.

Red ginseng is produced by steaming and drying *P. ginseng*. During the processing, ginsenosides in *P. ginseng* form other compounds with special physiological activities [55]. Hwang et al. found that red ginseng treatment would promote fat metabolism during exercise in mice to delay peripheral fatigue [37]. It has been found that fat oxidation was significantly higher in red ginseng treated mice during the initial 20 min of the one-hour aerobic running exercise, and the liver glycogen storage immediately decreased after the exercise in mice without red ginseng treatment. Moreover, free fatty acid (FFA) concentrations decreased in all mice rested for an hour after exercise, but there was no statistical difference among any groups at any time either. Therefore, a glycogen-sparing effect was observed in red ginseng-treated mice after one-hour exercise.

Besides delaying peripheral fatigue, a study showed that red ginseng was more effective in relieving psychological fatigue than physical fatigue [38]. Mice with red ginseng treatment had improved performance in series of behavioral experiments, including the electric field test, locomotor activity, the rotarod test, the balanced wire test, exploring an elevated plus maze, stress-related rearing behavior and the swimming test. Among them, stress significantly decreased locomotor activity in the open-field and exploratory activity in the elevated plus maze and rearing frequency, but red ginseng treatment can reverse it. Fear and anxiety increase corticosterone levels in the blood [56]. Moreover, red ginseng treatment can inhibit the release of corticosterone after exposure to restraint stress. However, red ginseng treatment did not change BLA content in this study. The decreased corticosterone level indicated that the red ginseng might relieve fatigue through the hypothalamic-pituitary-adrenal axis.

#### 3.1.2. In Chronic Fatigue Syndrome

Chronic fatigue syndrome (CFS) is characterized by persistent and unexplained fatigue resulting in severe impairment in the neuroendocrine system, cognitive and immune functions [57]. Today, it has been shown that *P. ginseng* has a positive effect on the treatment of CFS in animal models.

Cao et al. had preliminary evidence that KXS may ameliorate CFS by affecting the physiological parameters of fatigue [42]. In this study, CFS mice were created by forced wheel running for four weeks. After four weeks of exercise, KXS treated mice showed better performance in the running wheel test. In addition, the levels of BUN, lactic acid and LDH activity in muscle increased, and the levels of testosterone, glycogen in the liver and muscle decreased in CFS mice without KXS treatment. However, the intervention of KXS can inhibit the change of these indexes. In this study, after KXS treatment, splenocyte proliferation and secretion of IL-4 of splenocytes of mice significantly increased, and the abnormal excretion of IL-2 led by CFS decreased, which suggested that CFS may not only affect the balance of the immune system but also improve it in CFS mice through immunomodulation.

Wang et al. proved the mechanism of WGPA for the treatment of CFS [35]. In this study, WGPA was divided into the neutral polysaccharide fraction (WGPA-N) and more purified acidic polysaccharide fraction (WGPA-A). The mice treated with WGPA-A had the best performance in the forced swimming test. In further serum analysis, the increased content of MDA and LDH activity and the decreased activity of GPx and SOD had significantly reversed in WGPA and WGPA-A groups. In the meantime, morphological changes of mitochondria in striated skeletal muscle showed that WGPA-A treatment can clearly restore mitochondrial damage. Some studies reported abnormalities of mitochondrial structure in patients suffering from CFS [58,59]. These morphological changes of mitochondria further confirmed the mechanism of oxidative stress in CFS and the efficacy of WGPA-A in CFS treatment.

#### 3.1.3. In Cancer-Related Fatigue

Fatigue is one of the most common complications in cancer patients receiving cytotoxic chemotherapy, radiation therapy, bone marrow transplantation, or treatment with biologic response modifiers [60,61]. At present, there are no available therapeutic interventions approved by FDA [62]. However, some researchers had demonstrated that *P. ginseng* may be a kind of natural product treating cancer-related fatigue (CRF) in animal models.

Park et al. systematically examined BST204 (a kind of purified dry extract from *P. ginseng*) alleviating cancer chemotherapy-related fatigue in mice [36]. The BST204 treated mice had better performance in running wheel activity and forced swimming tests than mice without treatment. BST204 can significantly increase muscle glycogen levels and decrease the release of peripheral proinflammatory cytokines, including TNF-α and IL-6 stimulated by cancer and chemotherapy. The decreased cytokine levels supported one of the ideas that the effect of BST204 against CRF may through regulating inflammatory systems. In addition, the activity of ALT, AST and creatinine (indicator for evaluating kidney damage, Cr) level were markedly decreased with high dose BST204 treated. It was reported that anemia in cancer patients might contribute to CRF, and the increased symptoms of fatigue correlated to abnormally low levels of hemoglobin [36]. High dose BST204 also can significantly increase levels of white blood cell (WBC), neutrophil (NEUT), red blood cell (RBC), and hemoglobin (HGB) compared with the control group. The improvement of hematologic parameters also suggested that the effect of BST204 on CRF may be explained by its promotion of hematopoiesis.

### 3.2. The Anti-Fatigue Effects of Monomer Compounds

#### 3.2.1. In Normal Fatigue

The ginsenosides can be classified as protopanaxadiols or protopanaxatriols. Orally administered protopanaxadiol-type and protopanaxatriol-type ginsenosides are metabolized to 20 (S)-protopanaxadiol (PPD) and 20 (S)-protopanaxatriol (PPT) [63,64,65,66]. Oh et al. found that the ginsenoside metabolites had anti-fatigue activity [43]. The results showed that PPT can significantly prolong both swimming time in forced swimming tests and running time in rotarod tests. Besides improving performance in fatigue-related behavioral experiments, PPT can also markedly increase glucose levels and decrease levels of corticosterone, BLA, FFA, Cr and LDH activity. Interestingly, PPD had a similar effect on biomarkers compared to PPT but did not improve the exercise endurance in a behavioral test, which suggested that PPT was more potent than PPD. These studies all suggest that the anti-fatigue effect of *P. ginseng* was attributable to the ginsenosides, which can be metabolized into PPT.

Chen et al. considered that one of the protein targets of PPD in skeletal muscle tissue was muscle-type creatine kinase (CK-MM) [44]. In addition, PPT and ginsenoside Rh2 also can show the same tendency to the mouse CK-MM activity. Moreover, excessive PPD (20 μM) may have adverse effects on CK-MM activity. In addition to increasing CK-MM activity, PPD treatment also can upregulate skeletal muscle phosphocreatine level in vivo. The slightly improved muscle CK-MM activity and phosphocreatine level can reduce lactate accumulation and substantially improve the performance in forced swimming tests. There were three possible mechanisms of the CK/phosphocreatine system proposed in this study. First, high phosphocreatine level, phosphocreatine/ATP ratio and CK-MM activity increased energy storage and synthesis of ATP, which were crucial for skeletal muscle [67]. Additionally, the ATP-protective function of phosphocreatine might relieve fatigue by delaying the occurrence of glycolysis. Second, the improvement of the CK/phosphocreatine system can be beneficial to relieve metabolic acidosis. Finally, the enhancement of CK-MM activity may counteract the decreased intracellular CK-MM activity caused by muscle tissue damage, keeping the original CK-MM activity in the cell to eliminate fatigue.

Shin et al. reported the anti-fatigue properties of panaxydol (a kind of active component in cultivated wild ginseng) in rats [45]. In behavioral experiments, panaxydol can significantly prolong forced swimming time. However, no difference was found for the level of liver and soleus muscle glycogen between the panaxydol group and the control group. Although no marked change was observed for BUN and BLA levels, panaxydol can decrease LDH level, which increased during forced swimming tests. All the results showed that panaxydol can enhance forced swimming performance only by changing the LDH level. However, the mechanisms of panaxydol reducing LDH activity and muscle damages were not clear in this study.

#### 3.2.2. In Postoperative Fatigue Syndrome

Postoperative fatigue syndrome (POFS) is a complication, which commonly happens after surgery, especially major abdominal and cardiac procedures [68]. Patients affected by POFS often feel malaise, lethargy, energy loss, distraction, and asthenia [69,70]. Meanwhile, POFS also impede postoperative recovery, decline the maximum force and reduce muscle endurance, which can burden patients greatly [71]. As a kind of effective anti-fatigue botanical medicine, *P. ginseng* can also relieve POFS.

Ginsenoside Rb1, an important active compound in *P. ginseng*, can resist POFS induced by major small intestinal resection in rats [46]. Although no differences were found for the BUN level in all the groups, Rb1 can raise not only the maximum grip strength but also reverse the increased BLA level and LDH activity. In addition, the postoperative rats treated by Rb1 showed higher muscle glycogen level and SOD activity with a lower MDA level. Fortunately, Rb1 treatment would not change the muscle microstructure. Meanwhile, Rb1 can improve the energy metabolism in the skeletal muscle [72]. In this study, Rb1 showed the capacity to increase locomotor activity and total food intake of POFS rats. In addition, Rb1 can increase ATP content and Na^+^-K^+^-ATPase activity, as well as keep the succinate dehydrogenase (SDH) activity continuously higher after surgery. However, no differences were found for blood glucose level and muscle pyruvate kinase (PK) activity in all the rats. As one of the main ATP degradation enzymes, Na^+^-K^+^-ATPase can hydrolyze ATP to supply direct free energy [73]. Na^+^-K^+^-ATPase might be one of the crucial factors associated with fatigue [74]. Moreover, SDH and PK are two rate-limiting enzymes for catalyzing ATP synthesis, which is related to regulating the glycolytic pathway and the Krebs cycle, respectively [75]. Therefore, all the results suggested that Rb1 may be an effective component to regulate the decreased activity of the energy metabolism-related enzymes caused by the oxidative stress damage in POFS [73].

It was reported that ginsenoside Rb1 may improve POFS through the activation of the PI3K/Akt/Nrf2 pathway [47]. In this study, it was found that Rb1 can raise the journey and feeding frequency of the POFS rats in open field tests and increase SOD activity, as well as reduce MDA and ROS levels. In addition, Rb1 can also upregulate the expression of Akt and Nrf2 mRNA in skeletal muscle. The Western blot analysis showed similar results that Rb1 treated rats had higher levels of phosphorylated Akt and nuclear Nrf2. As for the upregulation of phosphorylated Akt, some studies proved that the upregulation of nuclear Nrf2 demanded the activation of the PI3K/Akt pathway, which can induce the phosphorylation of Akt [76,77]. All these findings supported that the effect of Rb1 on POFS rats might depend on the activation of the PI3K/Akt pathway with Nrf2 nuclear translocation.

Ginsenoside Rg3, another active compound of *P. ginseng*, was considered to have the fatigue resistance effect on POFS rats [48]. Rg3 can also increase journey and feeding frequency. In addition, Rg3 can increase the level of TC and TG, increase LDH and SOD activity and decrease MDA levels in muscles. These findings demonstrate that the fatigue resistance effects of Rg3 might be related to inhibition of lipid peroxidation. The increased TC and TG levels suggest that Rg3 treatment may delay fatigue symptoms by better utilizing fatty acid to spare more glycogen and glucose [78,79]. In this study, Rg3 was able to upregulate the expression of PGC-1α and phosphoenolpyruvate carboxykinase (PEPCK), increase the silent information regulator of transcription 1 (SIRT1) deacetylase activity and decline the transcriptional activity of p53. PEPCK was a kind of gluconeogenic gene regulated by the expression of PGC-1α [13,80]. Clearly, SIRT1 can improve exercise performance by enhancing mitochondrial activity [81]. Moreover, SIRT1 was related to oxidative stress response because of the deacetylation of p53 and other transcription factors [82,83]. In summary, Rg3 may play an anti-fatigue and antioxidative role probably mediated by activation of SIRT1.

## 4. Anti-Fatigue Effect in the Clinical Setting

The common clinical manifestations of fatigue are a state of tiredness, weakness, and exhaustion with the feeling of weariness, sleepiness, irritability, and cognitive impairment [84]. Unlike normal fatigue, pathogeny and symptom of pathological fatigue were more complex. Take cancer-related fatigue as an example. There was the criterion drafted by the Fatigue Coalition in 1998 for diagnosing the severity of fatigue was divided into multiple symptoms, including the increased need to rest, generalized weakness or limb heaviness, diminished concentration, decreased motivation, insomnia, or hypersomnia, nonrestorative sleep, emotional reactivity (e.g., sadness, frustration or irritability), fatigued completing daily tasks, perceived problems or post-exertional malaise lasting several hours [85].

Nowadays, various scales were used in clinical trials to evaluate fatigue. For example, visual analog fatigue scale (VAFS) can capture an aspect of fatigue, typically severity or intensity, through a 100 mm horizontal line fixed by two statements representing extreme ends of a single fatigue continuum, which can describe the patients’ fatigue status, such as “no fatigue” to “extreme exhaustion” or “complete fatigue” [86]. Multidimensional fatigue scale (MFS), which contained 29-item fatigue assessment instrument, including physical, psychological, and social symptoms, can objectively measure the degree of fatigue [87]. Moreover, a self-rating numeric scale (NRS) is a questionnaire consisted of seven physical health-related questions and four mental health-related questions to reflect more fatigue with lower scores [88]. In addition, the brief fatigue inventory (BFI), which consists of nine items to ask the patients how fatigued or tired they felt during the last week, was one of the questionnaires developed for rapid assessment of CRF with higher scores represent more severe fatigue [89,90]. There are also some scales used to evaluate fatigue in specific diseases, such as the modified fatigue impact scale (a 21-item questionnaire, MFIS), which is used to evaluate multiple sclerosis (MS)-induced fatigue, and the Iranian version of the multiple sclerosis quality of life questionnaire (MSQOL) was used to estimate quality of life in MS patients [91,92]. While these scales show good results in fatigue evaluation, some other scales have also been tried to estimate fatigue degree. For example, the five-level EuroQol-5 Dimension (EQ-5D 5 L) had two parts to evaluate the health-related quality of life, mobility, self-care, usual activities, pain/discomfort, and anxiety/depression [93,94]. Stress response inventory (SRI)-short form and Beck depression inventory (BDI) can measure stress responses and symptoms of depression, respectively [95,96]. Sometimes, the various scales are also combined to determine the degree of fatigue more accurately.

The anti-fatigue effects of *P. ginseng* have been fully proved in animal models. However, few studies showed the anti-fatigue effects of *P. ginseng* in clinical trials. The anti-fatigue clinical studies of *P. ginseng* are summarized, and detailed clinical trials protocol are shown in Table 3.

### 4.1. In Normal Fatigue

Strenuous exercise can cause skeletal muscle damage and lead to a change of several parameters, such as CK in plasma and IL-6 [109,110]. It was reported that muscle contraction can release IL-6, an iconic cytokine, to measure the degree of muscle inflammation [111,112]. *P. ginseng* treatment can relieve fatigue and fatigue-related damage in college students caused by high-intensity uphill treadmill running tasks [97]. *P. ginseng* supplementation can decrease the elevated CK activity and IL-6 level, as well as reduce muscle damage. In addition, *P. ginseng* intake can improve insulin sensitivity by decreasing plasma glucose and insulin responses, which provided indirect evidence of blunted muscle damage. Therefore, the anti-fatigue effect of *P. ginseng* extract may be related to the antioxidative and anti-inflammation effects.

High production of some ginsenosides, such as Rh1, Rg3, compound K and PPT constituents, were found in Enzyme-modified ginseng, one of the processed ginseng products [113]. Lee et al. compared the constituent difference of standard ginseng and enzyme-modified ginseng and verified the anti-fatigue effect of enzyme-modified ginseng. The VAFS scores decreased in all the subjects during the trials, but the scores decreased more in subjects treated by enzyme-modified ginseng. Furthermore, there was no significant difference in other evaluating indicators. In addition, no significant differences were found for safety-related indicators, including AST, ALT, BUN, and Cr between the groups, which suggested that enzyme-modified ginseng was safe with no adverse effect in participants.

Another processed ginseng product (GBCK25), a kind of fermented ginseng powder, was produced by multiple fermentations, which can convert several ginsenosides to compound K [114]. It has been reported that high dose GBCK25 can markedly reduce MFS scores with the improvement of liver function [98]. Oxidative damage and inflammation of hepatocytes would affect liver function, which was involved in lipid and glycogen metabolism and affect energy production further. The results in this study suggested that GBCK25 can alleviate fatigue through improving liver function, which may be due to the fact that ginsenosides or compound K in GBCK25 can reduce the injury caused by oxidative stress and inflammatory response.

Metabolomics, a promising new technique for discovering novel biomarkers, can explore the potential mechanisms of diverse diseases [115]. Yan et al. investigated the metabolic pattern of athletes after *P. ginseng* treatment and explored the potential mechanism of *P. ginseng* on anti-fatigue effect through metabolomics [99]. In total, 11 types of metabolites (glyoxylate, 3-hydroxybutyrate, 3-methyl-2-hydroxybutyrate, suberyl glycine, 9-hexadecenoic acid, ribose, mannose, myoinositol, and 3 unknown metabolites) were identified in this study. After 30 days of *P. ginseng* treatment, the levels of 3-hydroxybutyrate, 9-hexadecenoic acid, suberyl glycine, ribose, and 3 unknown metabolites increased, and the levels of other 4 metabolites were decreased. As an unsaturated fatty acid, catabolism of 9-hexadecenoic acid can provide energy for the human body. 3-hydroxybutyrate was one of the products of fatty acid metabolism in the liver, which was an essential energy source of extrahepatic tissues associated with the synthesis of cholesterol, fatty acids, or complex lipids. Glyoxylate was a product related to the change of fatty acid to provide energy. In addition, suberyl glycine was a kind of biomarker in diagnosing lipid oxidation dysfunction in mitochondrion [116]. Furthermore, ribose played an important role in the production and degradation of ATP. All of these metabolites were associated with lipid and energy metabolism.

### 4.2. In Pathological Fatigue

#### 4.2.1. In Chronic Fatigue

Like other kinds of chronic fatigue, idiopathic chronic fatigue (ICF) was more prevalent than CFS in the general US subjects [117]. Kim et al. used NRS and VAFS to monitor the fatigue severity combined with several biomarkers in ICF subjects [101]. The results showed that high dose *P. ginseng* can significantly decrease the VAFS scores. The total NRS scores had not decreased significantly after administration of all dosages. However, the mental NRS scores (a part of total NRS scores) involved in concentration, slips of tongue and clarity of thinking were significantly improved after *P. ginseng* treatment. In addition, *P. ginseng* treatment can significantly reduce ROS and MDA contents in serum. Taken together, *P. ginseng* showed a positive effect on relieving ICF, and its underlying mechanisms may be related to the antioxidative activity of *P. ginseng*.

Sung et al. found that red ginseng can reduce moderate CFS in patients older than 50 years [102]. Although the VAFS scores in red ginseng-treated subjects were lower, the score gap was not statistically significant. The SRI-short form, BDI, and EQ-5D 5 L showed a similar tendency to VAFS. No significant differences were observed in the change of biomarkers. Only the VAFS scores in the patients older than 50 years old or the patients with initial fatigue VAFS below 80 mm decreased significantly. The tendency of the results suggested that red ginseng may be useful to a part of chronic fatigue patients with specific conditions, such as advanced age or initial fatigue. In addition, red ginseng may be effective to the CFS for a long-term treatment duration because the subjects in this study were only treated for six weeks.

#### 4.2.2. In Cancer-Related Fatigue

Yennurajalingam et al. evaluated the effects of *P. ginseng* on CRF using multiple tools, including Edmonton Symptom Assessment System (ESAS), Functional Assessment of Chronic Illness Therapy-Fatigue (FACIT-F), hospital anxiety and depression scale (HADS), Global Symptom Evaluation (GSE), 6 min walk test (6MWT) and handgrip strength (HGS) [103]. It was found that *P. ginseng* intake can significantly reduce the severity of CRF in advanced cancer patients. However, there were no significant differences between the *P. ginseng* intake group and the placebo group in 29 days showed by the FACIT-F subscale. According to the results measured by all the tools, *P. ginseng* does not significantly improve quality of life, anxiety, depression, cancer-related symptoms, the patient-reported benefit of treatment on CRF, or physical function scores compared with the placebo group. However, there were fewer adverse events in the *P. ginseng* group. Based on these outcomes, there was no justification for *P. ginseng* to be recommended for relieving CRF in advanced cancer patients.

Although there was no significant improvement of CRF in advanced cancer patients, the symptoms of CRF (pain, appetite and quality of life) can be markedly improved in the patients suffering from nonmetastatic colon cancer after ingesting *P. ginseng* for 30 days [104]. Particularly, chemotherapy and radiotherapy were major factors that can develop severe fatigue [118,119]. Red ginseng can moderate chemotherapy-induced fatigue in colorectal cancer patients, especially older and female patients [105]. Red ginseng can significantly reduce BFI scores over 8 weeks and 16 weeks. In addition, the improvement efficacy for fatigue over 16 weeks was greater than that over 8 weeks. Based on these findings, red ginseng was more effective in patients with a longer treatment period.

In conclusion, *P. ginseng* showed improvement in CRF, especially in the patients who were older or felt more fatigue. However, it is difficult to define the mechanism of *P. ginseng* on CRF according to existent studies since placebo also can reduce CRF in clinical studies. Thus more forceful evidence is needed to support using *P. ginseng* in clinical.

#### 4.2.3. In Other Pathological Fatigue

Zhang et al. estimated the safety and anti-fatigue effect of red ginseng in a randomized, double-blind, and placebo-controlled clinical trial [106]. The participants in this study were diagnosed with asthenia syndrome. Although the fatigue self-assessment scores decreased in all three groups after treatment, the scores of the red ginseng-treated group were decreased more than that of the placebo group. Moreover, fewer participants showed no alleviation of fatigue symptoms in the red ginseng-treated group. In addition, red ginseng treatment would not lead to any fire-heat symptoms (some adverse symptoms, such as chest or abdomen scorching, mouth ulcers, and constipation, even like hectic fever and insomnia [120]), which indicated that there was no safety concern with the intake of red ginseng.

Multiple sclerosis (MS) is the most common nontraumatic disabling disease to affect young adults with increased incidence and prevalence in both developed and developing countries [121]. One of the least known symptoms in MS patients is long-term fatigue, which reduces quality of life [122]. Etemadifar et al. suggested that *P. ginseng* would relieve fatigue symptoms in MS patients and enhance their quality of life after treatment for three months [107]. *P. ginseng* treatment can significantly improve the MFIS scores in the physical subscale part with no marked differences in cognitive and psychosocial parts. Moreover, most of the scores of subitems of the MSQOL questionnaire, such as physical health, energy, change in health and role limitations, were increased after *P. ginseng* intervention. The study implied that the antioxidative effect of *P. ginseng* can resist fatigue during MS and relieve the symptoms of MS because there have been some theories demonstrating the roles of oxidative stress and free radicals in the pathogenesis of MS [123,124].

It was reported that nonalcoholic fatty liver disease (NAFLD) patients suffered from a series of chronic fatigue with peripheral inflammation and immune activation [125,126]. Hong et al. showed that red ginseng can significantly decrease the severity of fatigue caused by NAFLD, especially in overweight NAFLD patients [108]. Red ginseng can significantly reduce the TNF-α levels in overweight NAFLD patients. Adiponectin was considered to be related to fatty liver disease [127]. Moreover, red ginseng treatment increased serum adiponectin levels, which suggested that red ginseng can positively affect NAFLD. All of the results indicated that red ginseng can relieve NAFLD and NAFLD-related fatigue, possibly because of the anti-inflammatory effect of red ginseng.

Diseases-related fatigue was generally caused by various acute and chronic inflammatory diseases, which greatly hindered the treatment of diseases. Reviewing all the studies targeted on diseases-related fatigue, *P. ginseng* was an effective supplementation in clinical treating diseases-related fatigue. For example, red ginseng can significantly improve mood, relations with others, walking ability and enjoyment of life in colorectal cancer patients with chemotherapy [105]. Oral ginseng also can improve quality of life, such as muscular pain, degree of happiness, appetite and sleep quality, as well as mood condition in nonmetastatic cancer patients [104]. In general, *P. ginseng* treatment can improve the quality of life in patients with various diseases, which was better in long-term treatment. However, *P. ginseng* was not effective in all types of disease-related fatigue. Fatigue-related symptoms, such as emotion, tiredness, sleep quality and self-feeling of wellbeing, were not improved in advanced cancer patients after *P. ginseng* treatment [103]. In general, *P. ginseng* can improve physical and mental conditions in most instances. However, there were differences in the curative effect of different diseases.

## 5. Conclusions

This review summarized the anti-fatigue effects of *P. ginseng* in animal models and clinical trials. Several anti-fatigue compounds and possible mechanisms were also discussed in this review. Abundant studies showed that *P. ginseng* can enhance animals’ exercise endurance with the change of biomarkers, especially BUN, MDA, SOD, and BLA. In addition, advanced research has demonstrated that *P. ginseng* can regulate the expression of proinflammatory cytokines (IL-6, TNF-α, IL-1β) and activate oxidative stress-related pathways, such as Nrf2-ARE and PI3K/Akt signaling pathway, which suggested *P. ginseng* may perform anti-fatigue effects through antioxidation, anti-inflammatory activity, reduction of metabolites accumulation, or management of energy metabolism. *P. ginseng* also had the effects of fatigue resistance in clinical patients suffering from multiple different diseases. However, this effect was not sufficiently clear. Thus, further study is needed to find clear evidence in clinical application and specific mechanism of *P. ginseng* in anti-fatigue effects.

## Figures and Tables

**Table 1:** Anti-fatigue effects of mixture extract from *P. ginseng* in animal models.

Materials	Compounds	Classification	Animal Model	Effects	Reference
*P. ginseng*	Water-soluble polysaccharides	Normal fatigue	Male ICR mice	Enhanced swimming time Increased SOD and GPx activity, glucose levels Decreased LDH and CK activity, TG and MDA levels	Wang et al. [32].
	Extracts	Normal fatigue	Male ICR mice	Enhanced forelimb grip strength, swimming time Increased glucose, total protein, albumin and muscle glycogen levels Decreased BLA, NH_3_, BUN and TG levels, CK activity	Ma et al. [33].
	GOP	Normal fatigue	Male ICR mice	Enhanced swimming time Increased liver glycogen level, LDH, SOD and catalase activity Decreased BUN, MDA and BLA levels Increased expression of NRF-1 and TFAM	Bao et al. [34].
	Water-soluble polysaccharides	CFS	Male ICR mice	Enhanced swimming time Increased SOD and GPx activity Decreased LDH activity and MDA levels	Wang et al. [35].
	BST204 (purified dry extract)	Cancer chemotherapy-related fatigue	Female BALB/c-nu/nu mice	Enhanced running wheel activity, swimming time Increased muscle glycogen, WBC, NEUT, RBC and HGB levels Decreased ALT and AST activity, Cr, TNF-α and IL-6 levels	Park et al. [36].
Red ginseng	Extracts	Peripheral fatigue	Male ICR mice	Enhanced liver glycogen storage Accelerate fat oxidation	Hwang et al. [37].
		Psychological fatigue	Male ICR mice	Improved performance in electric field test, locomotor activity, rotating rod test, balanced wire test, exploring elevated plus maze, stress-related rearing behavior and swimming test Inhibited release of corticosterone	Choi et al. [38].
*P. ginseng* with notoginseng	Extracts	Normal fatigue	Male Kunming mice	Enhanced swimming time Increased liver glycogen levels Decreased BLA, BUN levels	Chen et al. [39].
*P. ginseng* with *Antrodia camphorata*	Extracts	Normal fatigue	Male ICR mice	Enhanced swimming time and forelimb grip strength Increased glucose and muscle glycogen levels Decreased BUN and ammonia levels, CK activity	Hsiao et al. [3].
*P. ginseng with Acanthopanax senticosus*	Extracts	Normal fatigue	Male ICR mice	Enhanced swimming time Increased liver glycogen level, LDH, GPx and SOD activity Decreased BUN levels	An et al. [40].
Kai Xin San	Extracts	Normal fatigue	Sprague-Dawley (SD) rats	Enhanced running time Increased muscle and liver glycogen and testosterone levels, SOD activity Decreased BUN, BLA, β-endorphin and MDA levels	Hu et al. [41].
	Extracts	CFS	Male Kunming mice	Improved performance in running wheel test Increased muscle and liver glycogen, testosterone levels Decreased MDA and BUN levels, LDH activity Increased IL-4 levels Decreased IL-6 levels	Cao et al. [42].

**Table 2:** Anti-fatigue effects of monomer compounds from *P. ginseng* in animal models.

Compounds	Classification	Animal Model	Effects	Reference
20(S)-protopanaxatriol	Normal fatigue	Male ICR mice	Improved performance in swimming test and rotarod test Increased glucose levels Decreased corticosterone, BLA, FFA, Cr levels and LDH activity	Oh et al. [43].
	Normal fatigue	Male ICR mice	Increased CK-MM activity	Chen et al. [44].
20(S)-protopanaxadiol	Normal fatigue	Male ICR mice	Increased glucose levels Decreased corticosterone, BLA, FFA, Cr levels and LDH activity	Oh et al. [43].
	Normal fatigue	Male ICR mice	Enhanced swimming test Increased CK-MM activity, muscle phosphocreatine and ATP levels Decreased MLA levels	Chen et al. [44].
Ginsenoside Rh2	Normal fatigue	Male ICR mice	Increased CK-MM activity	Chen et al. [44].
Panaxydol	Normal fatigue	Male SD rats	Enhanced swimming time Increased LDH activity	Shin et al. [45].
Ginsenoside Rb1	Postoperative fatigue syndrome	Male SD rats	Enhanced grip strength Increased muscle and liver glycogen level, ATP, Na^+^-K^+^-ATPase, SDH, LDH and SOD activity Decreased MDA and BLA levels	Tan et al. [46].
	Postoperative fatigue syndrome	Aged male SD rats	Improved performance in open field test Increased SOD activity Decreased MDA, ROS levels Increased expression of Nrf2 and Akt	Zhuang et al. [47].
Ginsenoside Rg3	Postoperative fatigue syndrome	Aged male SD rats	Improved performance in open field test Increased TC, TG levels and LDH, SOD, SIRT1 activity Decreased MDA levels and transcriptional activity of p53 Increased expression of PGC-1α and PEPCK	Yang et al. [48].

**Table 3:** Anti-fatigue of *P. ginseng* in clinical trials.

Classification	Study Design	Participants (Total/Final)	Age (Years)	Placebo Group	Intervention Group	Effects on Fatigue	Reference
Normal fatigue	Single-blind—7 days before exercise and 3 days after exercise	18/18 Male college students	19.9 ± 0.6 (placebo group) 20.2 ± 0.5 (red ginseng group)	200 mL × 0.02 g mL^−1^ Agastachis Herba tea 3 times per day (*n* = 9)	200 mL × 0.1 g mL^−1^ red ginseng extract 3 times per day (*n* = 9)	Decreased CK activity and IL-6 level Improved insulin sensitivity	Jung et al. [97].
	Double-blind—12 weeks	90/84 People with serum ALT level of 35–105 IU/L	43.52 ± 11.02	1.4 g d^−1^ placebo	1.4 g d^−1^ powder with 125 mg d^−1^ fermented ginseng (low dose) 1.4 g d^−1^ powder with 500 mg d^−1^ fermented ginseng (high dose)	Decreased MFS scores	Jung et al. [98].
	Single-blind—30 days	21/21 Professional players	22 ± 3 (placebo group) 24 ± 5 (treatment group)	500 mg d^−1^ placebo	500 mg d^−1^ Korean ginseng powder	Increased testosterone level Increased 3-hydroxybutyrate, 9-hexadecenoic acid, suberyl glycine, ribose and 3 unknown metabolites levels Decreased glyoxylate, 3-methyl-2-hydroxybutyrate, mannose and myoinositol levels	Yan et al. [99].
	Double-blind—4 weeks	52/47 Healthy adults	60.1 ± 4.44 (placebo group) 62.1 ± 5.18 (treatment group)	Two placebo capsules twice a day	Two enzyme-modified ginseng extract capsules twice a day (2000 mg d^−1^)	Decreased VAFS scores more No adverse effect	Lee et al. [100].
ICF	Double-blind—4 weeks	90/88 Adults with ICF	39.5 (median age, 20–60)	Four placebo capsules (250 mg each) twice a day	Four capsules (250 mg each) twice a day, 1 g *P. ginseng* totally Four capsules (250 mg each) twice a day, 2 g *P. ginseng* totally	Decreased VAFS scores and levels of ROS and MDA	Kim et al. [101].
CFS	Double-blind-treated for 6 weeks and followed up 4 weeks	50/47 Chronic fatigue patients	47.09 ± 10.80 (placebo group) 49.00 ± 8.35 (treatment group)	3 g d^−1^ placebo	3 g d^−1^ Korean red ginseng powder	Attenuated VAFS scores, SRI-short form, BDI and EQ-5D 5 L, but no statistically decreasing	Sung et al. [102].
Cancer-related fatigue	Double-blind—4 weeks	127/112 Advanced cancer patients	61.0 (median age, 54.0–67.0)	400 mg d^−1^ placebo twice a day	400 mg d^−1^ *P. ginseng* twice a day	No statistically differences between the two groups	Yennurajalingam et al. [103].
	Single-blind—30 days	114/113 Nonmetastatic colon cancer patients	48.03 ± 10.56 (placebo group) 50.11 ± 10.46 (treatment group)	100 mg d^−1^ placebo	100 mg d^−1^ *P. ginseng*	Improved quality of life and appetite Ease pain	Pourmohamadi et al. [104].
	Double-blind—16 weeks	429/348 colorectal cancer patients	60 (median age, placebo group, 27–86) 60 (median age, treatment group, 29–84)	500 mg × 2 pills placebo twice a day	500 mg × 2 pills Korean red ginseng twice a day	Decreased BFI scores	Kim et al. [105].
Diseases-related fatigue	Double-blind—4 weeks	180/174 Asthenia syndrome volunteers	36.13 ± 11.35	Placebo capsules each day	1.8 g/3.6 g Korean red ginseng each day	Decreased fatigue self-assessment scores and TCM symptom scores No increased fire-heat symptom scores and abnormalities	Zhang et al. [106].
	Double-blind—3 months	52/52 multiple sclerosis patients	34.5 ± 8.9 (placebo group) 33.3 ± 7.5 (treatment group)	250 mg d^−1^ placebo twice a day	250 mg d^−1^ *P. ginseng* twice a day	Increased MSQOL scores Decreased MFIS scores	Etemadifar et al. [107].
	Single-blind—3 weeks	80/66 nonalcoholic fatty liver disease patients	47.8 ± 14.9	3000 mg d^−1^ placebo capsule	3000 mg d^−1^ Korean red ginseng capsule	Increased serum adiponectin levels Decreased TNF-α levels in overweight patients	Hong et al. [108].
